# Changes of Dynamic Functional Connectivity Associated With Maturity in Late Preterm Infants

**DOI:** 10.3389/fped.2020.00412

**Published:** 2020-07-23

**Authors:** Xueling Ma, Xiushuang Wu, Yuan Shi

**Affiliations:** ^1^Department of Neonatalogy, Children's Hospital of Chongqing Medical University, Chongqing, China; ^2^National Clinical Research Center for Child Health and Disorders, Chongqing, China; ^3^Key Laboratory of Child Development and Disorders, Ministry of Education, Chongqing, China; ^4^Department of Pediatrics, Daping Hospital, Army Medical University, Chongqing, China; ^5^Department of Pediatrics, Yunnan Provincial Crops of Chinese People's Armed Police Force, Langfang, China; ^6^Chongqing Key Laboratory of Child Infection and Immunity, Chongqing, China

**Keywords:** late preterm infants, premature brain injury, fMRI, independent component analysis, dynamic functional connectivity

## Abstract

**Objective:** To investigate the changes of dynamic functional connectivity (DFC) in late preterm infants, and assess whether these changes are associated with the indicators measuring the maturity of neonates.

**Methods:** Resting-state fMRI (rs-fMRI) data of eligible neonates was acquired with a 3.0-T MRI scanner in the Department of Radiology, Daping Hospital, Army Medical University (Chongqing, China). After the selection of functional connectivity networks obtained by independent component analysis (ICA), a sliding-window approach was used to cluster all the windows into different states. Then the differences of temporal properties of DFC between groups were compared, and the association between these temporal properties and the degree of maturity was also explored in each state.

**Results:** Eventually, 34 late preterm and 37 term neonates were included in the final analysis. Based on their data, 5 components were located in 5 networks: default-mode (DMN), dorsal attention (DAN), auditory (AUD), sensorimotor (SMN), and visual (VN). Then four reoccurring state patterns of functional connectivity were identified with the k-means clustering method. The late preterm group dwelled significantly longer in State III (late preterm: 33.57 ± 37.64 s, term: 18.50 ± 11.71 s; *P* = 0.03), which was characterized by general weaker connectivity between networks. Also, the correlation analysis shows the degree of maturity is negatively correlated to the dwell time and fractional windows in State III.

**Conclusion:** Our findings suggested that compared with term infants, late preterm infants preferred to stay in a state with general weak connectivity between networks, but this preference declined as maturity increased.

## Introduction

Preterm birth accounts for 11% of all live-births worldwide, and their survival rate has remarkably increased in recent few years (1, 2). Preterm infants, even late preterm infants (born between 34^+0^ and 36^+6^ weeks of gestation), are still at risk for neurodevelopmental impairment. Accounting for about 75% of all preterm births, late preterm infants are the largest group of preterm newborns. Severe brain injuries such as cerebral palsy seldom happen in late preterm infants, but mild to moderate injuries often occur in this group. According to recent studies, mild injuries may also lead to developmental and emotional-behavioral problems in adolescence, involving language disorder, attention deficit hyperactivity disorder, developmental coordination disorder, autism spectrum disorder, and so on (3, 4).

A series of characteristic alterations in the structural and functional connectivity of brain were identified in many neurological disorders. By observing these alterations, the associations across many brain disorders may be revealed (5). As for neurodevelopmental problems caused by premature birth, both commonalities and differences in clinical characteristics have been noticed. Therefore, exploring characteristic alterations of the connectivity networks in preterm infants may help reveal mechanisms of various disorders related to premature brain injury. For instance, lesions in different locations can cause similar symptoms if these lesions affect the same brain network. For this reason, we chose to analyze the functional network connectivity in late preterm neonates.

A recent technique called dynamic functional connectivity (DFC), assesses temporal variations of functional connectivity during MRI acquisition by dividing resting-state functional MRI (rs-fMRI) scans into a series of “sliding windows” (6, 7) and clustering these windows into several states by k-means method (6, 8).

We hypothesized that the temporal properties of DFC between term and late preterm infants were different, and there were associations between these properties and indicators measuring maturity.

## Materials and Methods

### Participants

A total of 155 neonates were initially recruited from the Department of Pediatrics, Daping Hospital, Army Medical University (Chongqing, China), including 58 term and 97 late preterm infants. Enrolled patients were initially chosen following these criteria: neonates without unstable medical condition or contraindication to MRI, gestational age more than 34 weeks, no acute or chronic diseases, no resuscitation history at birth, no major congenital malformations, and no congenital infections. After the acquisition of MRI data, two experienced experts reviewed the imaging and reported the result together, infants with definite or suspicious intracranial hemorrhage or other major structural abnormalities were also excluded in the following analysis. This step excluded 32 preterm and 3 term infants, remaining 120 infants in the dataset.

This study was approved by the Ethics Committee of Daping Hospital, Army Medical University (Chongqing, China). All study procedures followed the Declaration of Helsinki. Written informed consent was obtained from every infant's parents.

### Data Acquisition

Scans were collected using a 3.0-T MRI scanner (Siemens, Germany) during natural sleep in the Department of Radiology, Daping Hospital, Army Medical University (Chongqing, China). Infants were transported to MRI scanner, accompanied by a nurse and a neonatologist. Scanning was immediately done after the infant was fed to induce drowsiness. Neonatal ear muffs were used to block out MRI noise. During the examination, each participant was continuously monitored by an electrocardiogram and a pulse oximeter and closely observed by the accompanied neonatologist. Structural images were collected with a turbo-spin echo (TSE), T2-weighted sequence, TR/TE/flip angle = 3,200/393 ms/150°, voxel size = 1.25 × 1.25 × 1.95 mm^3^; 96 transversal slices, bandwidth = 751 Hz/pixel. Rs-fMRI scans were collected using a T2-weighted echo-planar imaging (EPI) sequence, field of view = 220 × 220 mm^2^, TR/TE/flip angle = 2,000/30 ms/90°, voxel size = 3.4 × 3.4 × 3.0 mm^3^ with no gap, number of slices = 33. For each neonate, 240 volumes were obtained across the whole brain.

### Preprocessing

**Figure 1:**
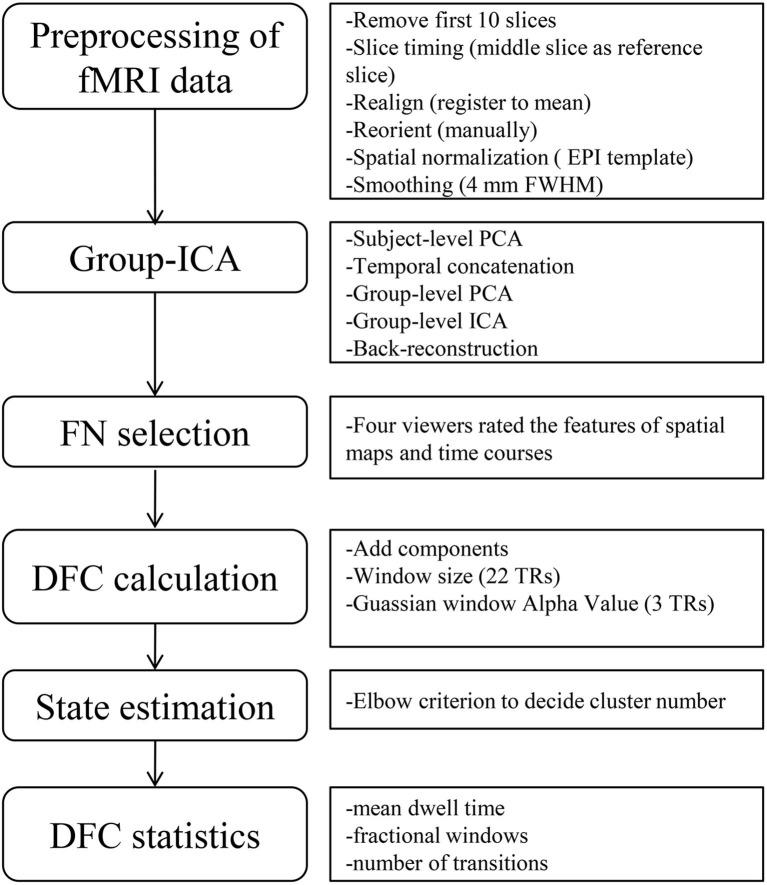
Flow chart of data processing. The left part shows the overall steps of data processing, and the right part is the detailed sub-steps, including some important parameters in each step. ICA, independent component analysis; FN, functional networks; DFC, dynamic functional connectivity; FWHM, full width at half-maximum; PCA, principal component analysis; TRs, times of repetition.

The rs-fMRI data was preprocessed using Gretna2.0 (9) implemented in MATLAB (version R2013b)/SPM12. At the beginning of preprocessing, the first 10 volumes were removed to reach a steady state, leaving 230 volumes for each infant. Slice timing (middle slice as reference slice) was carried out for correction of acquisition time delay between slices, and realignment (register to mean) was carried out for correction of head motion between volumes. Since excessive head motion can affect DFC analysis (10), conservative inclusion criteria were chosen to minimize head-motion bias, which means acquisitions with frame wise displacement (FD) >0.5 mm would be removed (11), as well as the ones with translational movement more than 2 mm or rotational movement more than 2°. According to these criteria, we excluded 31 late preterm infants and 18 term infants, and 34 late preterm and 37 term infants were included in the final analysis. The following steps included reorientation manually, spatial normalization with EPI template (12), spatially smoothing with a Gaussian kernel (full width at half-maximum of 4 mm). The flow chart of data processing is shown in Figure 1.

### Group Independent Component Analysis

After data preprocessing, a data-driven technique called spatial independent component analysis (ICA) was performed with GIFT (version 3.0b) (13, 14) to decompose the data into spatial independent components. Two steps for data reductions were run during the analysis, that is, the subject-level and group-level principal component analysis. ICA was performed under the component number from 14 to 30 to obtain stable infant networks (the number estimated by minimum description length criteria is 14), and at last the component number of 25 was chosen for its relatively stable and intact network profiles. To replicate the decomposed independent components, the Infomax ICA algorithm was repeated for 20 times in ICASSO (15, 16) and the aggregate spatial maps were generated. With the group ICA back reconstruction method (17), the subject-specific spatial maps and their corresponding time courses were then generated.

### Functional Networks Selection

**Figure 2:**
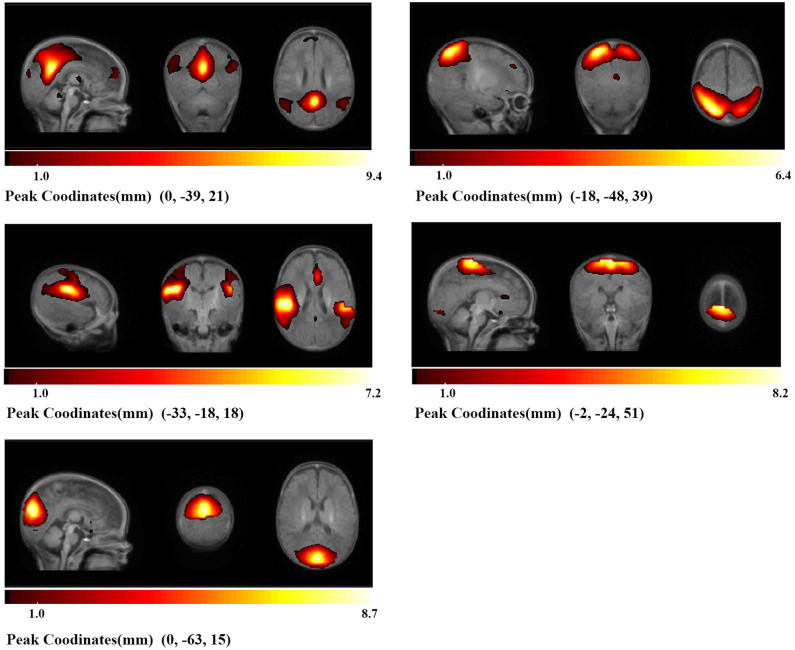
Identified networks. Based on the anatomical and functional properties, five components were located in five networks (DMN, DAN, AUD, SMN, VN). Brighter parts indicate stronger local activity, and under each part is its color bar and peak coordinates.

To differentiate between resting-state functional networks (FN) and physiological components, a previously described procedure was applied (18). Four viewers visually inspected the spatial maps and average power spectra, and scores from 0 (definite artifact) to 1 (certain functional network) were given by them based on these expectations: (1) RSNs should exhibit peak activation in gray matter, low spatial overlap with vascular, ventricular, and susceptibility artifacts; (2) Time course should be dominated by low-frequency fluctuations, with ratio of the integral of spectral power below 0.10 Hz to the integral of power between 0.15 and 0.25 Hz (19). Following these criteria, components were divided into 3 categories: artifact (score = 0), mixed (0 < score <3), functional network (score ≥ 3). This procedure resulted in 5 components located in 5 networks, default-mode (DMN), dorsal attention (DAN), auditory (AUD), sensorimotor (SMN), and visual (VN), shown in Figure 2.

Additional postprocessing of the selected components was performed following a previous research (6) to remove remaining noise sources. Briefly, steps included detrending, filtering with a cut-off value of low frequency fluctuation set as 0.15 Hz, and despiking.

### DFC Analysis

DFC was calculated based on a sliding window approach using GIFT toolbox. A sliding window length of 22 times of repetition (TRs) with a Gaussian window Alpha Value of 3 TRs was set, since this length could provide a good compromise between the quality of correlation matrix estimation and the ability to resolve dynamics (6). By sliding the window in a step length of 1 TR along the 230 TRs length scan, we obtained 208 consecutive windows across the entire scans. After computing DFC, all the functional connectivity matrices were transformed to *z*-score using Fisher's *z*-transformation for further analysis.

K-means method with sqEuclidean distance was used to regroup similar functional matrices of the different windows into different states, and the analysis was repeated 150 times to obtain a relatively stable result. The number of optimal clusters was calculated following the elbow criterion (6), and the number was set to be 4.

We investigated the temporal properties of DFC states derived from the state vector of every infant. Three measures in subjects were assessed, including: (1) Mean dwell time, defined as the average number of consecutive windows belonging to one state before changing to the other state; (2) fractional windows, defined as the number of total windows belonging to one state; (3) number of transitions, defined as the number of times that the state switched from one to the other.

### Statistical Comparisons and Correlations Analysis

The demographic and clinical data of all initially enrolled infants were recorded. Statistical analysis was performed using Statistical Package for Social Science 24.0 (SPSS 24.0). Differences in the demographic and clinical data were compared between the final included and excluded infants, and differences in maturity and temporal properties of DFC were compared between the late preterm and term infants. For continuous variables, we used Student's *t*-test for parametric data and Mann-Whitney *U*-test for nonparametric data. Pearson's chi-square test was used to compare categorical variables. We also carried out Pearson's correlation analysis between altered temporal properties and indicators measuring the maturity (birth weight, gestational age, postmenstrual age) for all participants. Significance was set at a *P* < 0.05 in all tests.

## Results

### Demographic and Clinical Characteristics

Recorded characteristics of all initially recruited infants were compared between the final included and excluded infants, including demographic characteristics such as gestational age, days of birth, and postmenstrual age when scanned, some maternal factors during pregnancy, and clinical data during hospitalization. No significant differences were found between them, as shown in Table 1. These results indicated that characteristics of the final included and excluded infants were roughly balanced. Then we compared the indicators measuring maturity between final included late term and term infants, which were significantly different between groups, including gestational age (preterm:35.93 ± 1.34, term:40.29 ± 1.06; *P* = 0.00), postmenstrual age when scanned (preterm: 37.28 ± 1.34, term:41.66 ± 1.13, *P* = 0.00), and birth weight (preterm: 2.03 ± 0.41, term: 3.27 ± 0.38, *P* = 0.00), as shown in Table 2.

**Table 1:** Demographic and clinical data of all initial enrolled patients.

	**Preterm**		**t/χ^2^**	***P***	**Term**		**t/χ^2^**	***P***
	**Included**	**Excluded**			**Included**	**Excluded**		
**Demographic data**								
Number	34	63			37	31		
Gestational age (weeks)	35.93 ± 1.34	35.64 ± 0.95	1.11	0.27	40.29 ± 1.06	40.00 ± 0.80	1.04	0.30
Birth weight (kg)	2.03 ± 0.41	2.03 ± 0.23	−0.05	0.96	3.27 ± 0.38	3.29 ± 0.39	−0.19	0.85
SGA infants	11/23	16/47	0.53	0.49	2/35	2/19	0.35	0.62
Delivery method (CS/VD)	9/25	15/48	0.08	0.81	13/24	5/16	0.80	0.40
Twins or triplets	2/32	2/61	0.41	0.61	2/35	0/21	1.18	0.53
Gender (male/female)	15/19	32/31	0.39	0.67	19/18	11/10	0.00	1.00
Days of birth (days)	9.47 ± 2.02	9.48 ± 2.06	−0.01	0.99	9.62 ± 2.30	9.62 ± 1.96	0.00	1.00
PMA (weeks)	37.28 ± 1.34	36.99 ± 0.95	1.10	0.28	41.66 ± 1.13	41.38 ± 0.92	0.96	0.34
**Maternal factors**								
Antenatal steroid	6/28	4/59	3.05	0.16	0	0		
PROM	1/33	2/61	0.00	1.00	3/34	1/20	0.23	1.00
DM, GDM, GH or PE	2/32	2/61	0.41	0.61	0/37	1/20	1.79	0.36
**Clinical data after birth**								
Apgar-1min	9.00 ± 1.07	9.24 ± 1.00	−1.09	0.28	9.38 ± 0.86	9.48 ± 0.51	−0.47	0.64
Apgar-5min	9.68 ± 0.48	9.83 ± 0.38	−1.57	0.12	10.00 ± 0.00	9.95 ± 0.22	1.00	0.32
Breast milk/mixed/formula	1/23/10	1/40/22	0.46	0.84	24/10/3	12/8/1	0.88	0.7
Ventilation time (hours)	25.76 ± 28.36	15.81 ± 25.67	1.76	0.08	0.00 ± 0.00	2.29 ± 10.47	−1.00	0.33
Apnea	2/32	2/61	0.41	0.61	0	0		
Sepsis	0	0			0	0		
Convulsions	0	0			0	0		
Hypothermia	1/33	1/62	0.20	1.00	0	0		

*Student's t-test or Mann-Whitney U-test was used for continuous variables, while Pearson's chi-square test was used for categorical variables. SGA, small for gestational age; CS, cesarean section; VD, vaginal delivery; PMA, postmenstrual age; PROM, premature rupture of the membranes; DM, diabetes mellitus; GDM, gestational diabetes mellitus; GH, gestational hypertension; PE, preeclampsia*.

**Table 2:** Demographic characteristics.

	**Late preterm**	**Term**	***t/*χ*^2^***	***P*-value**
Number	34	37		
Gestational age (weeks)	35.93 ± 1.34	40.29 ± 1.06	−15.27	0.00*
Days after birth (days)	9.47 ± 2.02	9.62 ± 2.30	−0.29	0.77
Postmenstrual age (weeks)	37.28 ± 1.34	41.66 ± 1.13	−14.88	0.00*
Birth Weight (kg)	2.03 ± 0.41	3.27 ± 0.38	−13.38	0.00*

Student's t-test or Mann-Whitney U-test was used for continuous variables, while Pearson's chi-square test was used for categorical variables. Significant results are marked with a *.

### Temporal Properties of Dynamic Connectivity in Each State

**Figure 3:**
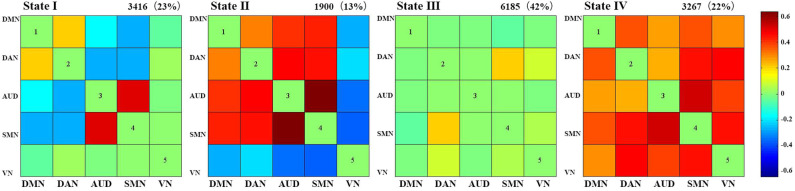
Cluster centroids of each state. The total number of occurrences and percentage of total occurrences of each state are listed above each cluster. State I was dominated by weak connections except the connection between SMN and AUD, State II was characterized by relatively strong positive and negative connectivity, State III was characterized by general weak connectivity between networks, State IV demonstrated relatively strong positive connections across nearly all networks.

We identified four reoccurring state patterns of functional connectivity based on the k-means clustering method. As illustrated in Figure 3, State III, which was characterized by general weak connectivity, occurred most frequently in all states (42%), while State II, which was characterized by relatively strong positive and negative connectivity, occurred least frequently (13%). More specifically, State III portrayed wide-spread weak between-network connectivity among all identified networks, including DMN, DAN, AUD, SMN, and VN, suggesting a rather static functional activity in State III. Additionally, State I occurred in 23% and State IV in 22% of all the windows. State IV demonstrated relatively strong positive connections across nearly all networks, whereas State I was dominated by weak connections except the connection between SMN and AUD.

As shown in Table 3, the mean dwell time in State I and State III was significantly different between groups. Specifically, the late preterm group showed a significantly shorter mean dwell time in State I (preterm: 10.36 ± 12.67, term: 25.43 ± 33.07, *P* = 0.02), while a significantly longer mean dwell time in state III (preterm: 33.57 ± 37.64, term: 18.50 ± 11.71; *P* = 0.03). Notably, as shown in the violin plot in **Figure 5B**, some infants in the late preterm group spent extremely long time in State III, while no one in the term group did. As for the fractional windows, significant differences between groups were also identified in State I and State III. No significant group differences were identified in the mean dwell time or fractional windows in State II and State IV. The number of transitions was not significantly different between groups, either.

**Table 3:** Temporal properties.

	**Preterm**	**Term**	***t***	***P***
Dwell time (State I)	10.36 ± 12.67	25.43 ± 33.07	−2.49	0.02*
Dwell time (State II)	14.83 ± 15.96	13.75 ± 34.18	0.17	0.87
Dwell time (State III)	33.57 ± 37.64	18.50 ± 11.71	2.24	0.03*
Dwell time (State IV)	22.12 ± 35.76	21.98 ± 47.00	0.01	0.99
Fractional windows (State I, %)	12.51 ± 17.24	32.89 ± 26.26	−3.89	0.00*
Fractional windows (State II, %)	13.05 ± 12.59	12.69 ± 20.56	0.09	0.93
Fractional windows (State III, %)	48.11 ± 25.69	36.16 ± 23.96	2.02	0.04*
Fractional windows (State IV, %)	26.33 ± 25.17	18.26 ± 26.34	1.32	0.19
Number of transitions	8.21 ± 3.91	8.57 ± 4.32	−0.37	0.71

For continuous variables, student's t-test was used for parametric data and Mann-Whitney U-test was used for non-parametric data, significant results are marked with a *.

**Figure 4:**
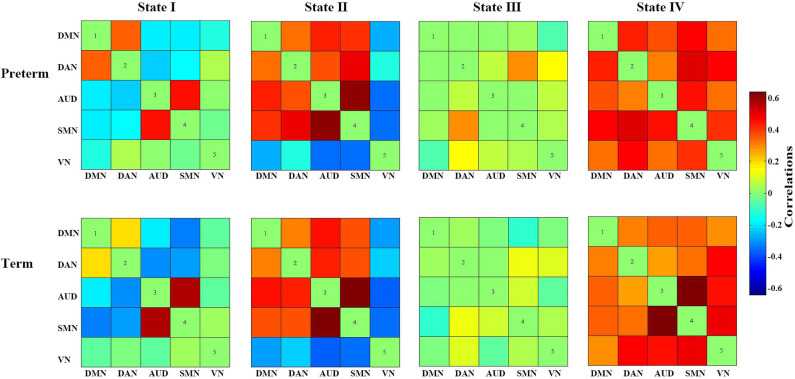
Between-group comparison of cluster centroids of each state.

**Figure 5:**
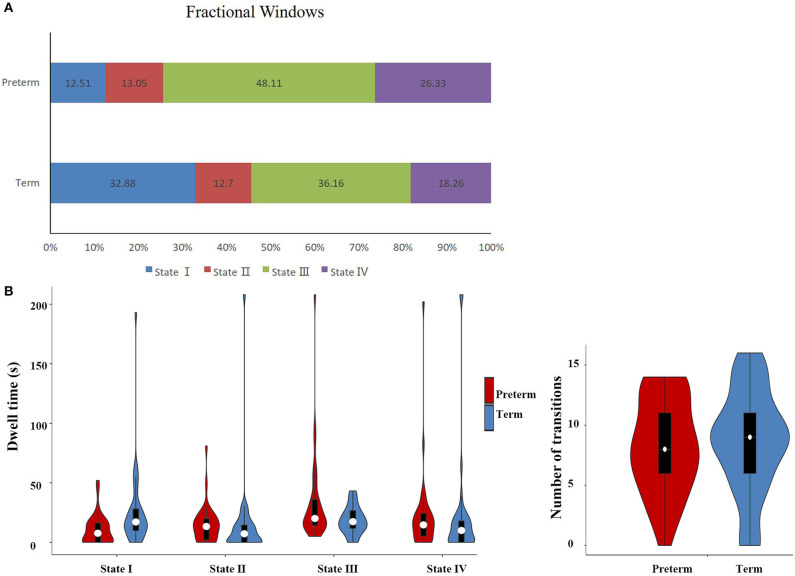
Temporal properties of DFC states for the late preterm and term neonates. **(A)** Percentage of total time that all subjects spent in each state. **(B)** Mean dwell time and number of transitions between states were plotted using violin plots. The fractional windows and mean dwell time in State I and State III were significantly different between groups. It is worth noting that some infants in the late preterm group spent very long time in State III, while no one in the term group did.

In summary, these changes indicated that the late preterm group switched states as frequently as the term infants, but they stay longer in State III than term infants. Since State III was characterized by general weak functional connectivity among all networks, it seemed that late preterm group preferred to stay in a more inactive state, compared with term neonates. Between-group comparison of cluster centroids of each state and the temporal properties of DFC were presented in Figures 4, 5, respectively.

### Correlation Between Clinical Data and DFC Properties

Correlation analysis was carried out to test whether the temporal properties of DFC were associated with the indicators measuring the maturity of included infants (gestational age, postmenstrual age, birth-weight). In line with our hypothesis, the mean dwell time and fractional time in State III showed a significant negative correlation with all these maturity indicators, whereas a positive correlation was found in state I, as shown in Table 4.

**Table 4:** Correlations between DFC temporal properties and clinical characteristics.

		**GA**	**PMA**	**BW**
Dwell time (State I)	*r*	0.370	0.406	0.383
	*P*	0.001*	0.000*	0.001*
Dwell time (State II)	*r*	−0.109	−0.109	−0.079
	*P*	0.367	0.367	0.511
Dwell time (State III)	*r*	−0.338	−0.327	−0.292
	*P*	0.004*	0.005*	0.014*
Dwell time (State IV)	*r*	0.043	0.015	−0.049
	*P*	0.724	0.898	0.685
Fractional windows (State I, %)	*r*	0.529	0.558	0.508
	*P*	0.000*	0.000*	0.000*
Fractional windows (State II, %)	*r*	−0.114	−0.120	−0.059
	*P*	0.343	0.319	0.623
Fractional windows (State III, %)	*r*	−0.279	−0.285	−0.219
	*P*	0.018*	0.016*	0.067
Fractional windows (State IV, %)	*r*	−0.151	−0.169	−0.227
	*P*	0.209	0.158	0.057
Number of transitions	*r*	0.059	0.045	0.084
	*P*	0.627	0.712	0.485

Pearson's correlation test was used, significant results are marked with a *. GA, gestational age; PMA, postmenstrual age; BW, birth-weight.

## Discussion

With increasing evidence emphasizing the neurodevelopmental problems faced by late preterm children (20), there is an increasing need to explore the possible underlying changes in their brains. Conventional brain MRI has the potential to detect even minor structural changes and help clinicians to make early diagnosis about premature brain injury. However, some late preterm infants without obvious structural changes also have developmental and emotional-behavioral problems in childhood and adolescence. Previous study has provided evidence for an aberrant structural and functional connectivity in preterm infants and a long-lasting impact of preterm birth on the organization of resting-state networks in school-aged children and adolescents (21, 22). Nonetheless, previous rs-fMRI studies of premature infants were mostly performed when they reached term equivalent age (23–25). As for the rapidly developing newborn brains, the best window for observing abnormalities may be missed. As far as we know, this is the first study that applied a DFC method to identify differences in the DFC properties between late preterm and term neonates, and the scanning time of all infants was from 34^+6^ to 43^+2^ weeks of postmenstrual age. Results demonstrated that a DFC approach can capture functional dynamics and reveal DFC characteristics in both term and late preterm brains across time.

In this study, we mainly focused on the temporal properties of the DFC, including the mean dwell time, fractional windows and the number of transitions. Besides, we investigated the association between these temporal properties and indicators measuring the maturity. Five networks (DMN, DAN, AUD, SMN, and VN) were found in included neonates, which demonstrates a much simpler constitution than the functional networks in adults. Based on identified networks, four connectivity states were found across all participants. Significant differences existed between groups in the time staying in State III, which was characterized by general weak connectivity. The result indicated the preference for a weak connectivity state in late preterm infants. In addition, the correlation analysis showed the degree of maturation was negatively correlated to the dwell time and fractional time in state III, whereas state I showed the opposite result. The late preterm group spent less time in State I, which was dominated by weak connectivity but relatively strong connectivity between SMN and AUD. However, cautions are needed when explaining the result about State I, since it contains negative connections, and the retest reliability of negative connections was questioned by a previous research (26).

Since functional connectivity of premature infants of different gestational age demonstrates different development stages out of the uterus, it is worth noting that in both groups, SMN shows a strong connectivity with AUD in all states except State I, which is in line with the regular sequence of neurodevelopment of this period. Previous studies have identified multiple RSNs incorporating cortical and subcortical gray matter regions, including those located in primary motor and sensory cortices (e.g., SMN, VN, AUD) and those involving association cortices (e.g., DMN, DAN, frontoparietal control) (23, 27, 28). It was reported that networks located in primary sensory and motor regions are established by term, and these networks demonstrate less variability between subjects (29). Our findings show that the connections between SMN and VN are strong in most of states in both late preterm and term group, which is consistent with the sequence of networks development identified by previous studies.

The causes and mechanisms of premature brain injury are so complex, so this study chose strict inclusion criteria to make it possible to investigate one of the possible mechanisms. In addition, this study excluded infants with hemorrhage to simplify the research condition and facilitate the spatial normalization process. At the same time, this inclusion criteria may cause bias for the analysis, since preterm infants have a significantly higher chance of intracranial hemorrhage than term infants, which usually occurs within 72 h after birth and can lead to long-term neurological sequelae (30). Besides, a unique challenge was placed in image registration in infants, especially in those with brain injury. Specifically, due to the rapid changes in the size and cortical folding of brain in early life, notable heterogeneity exists in the registration process when we process data from neonates of different maturity. Our study included patients whose postmenstrual age is between 34^+6^ weeks and 43^+2^ weeks, and used the neonatal atlas for both term and late preterm infants. A narrower period for gestational-age specific atlas, such as 2–4 weeks, might make the spatial normalization more precise. In addition, we found some infants in the late preterm group spent extremely long time in State III, so the follow-up of neurodevelopment in these infants is very meaningful for they might have a higher chance of neurodevelopmental problems. But our study failed to perform reliable statistical analysis of the follow-up data due to the low completion rate of follow-up. However, though there is a lack of direct evidences to support the view that the length of time in State III is positively associated with neurodevelopment in the future, indirect evidences provided by previous studies of static functional connectivity indicate that preterm birth has persisting developmental effects on functional connectivity and motor performance in children, and altered static functional connectivity is related to motor development in childhood (31, 32). In addition, studies of DFC in adults have shown that the dwell time in a weakly connected state is associated with cognitive and intellectual impairment in a variety of neuropsychiatric disorders such as Parkinson's disease, autism and depression (33–35).

To conclude, this is the first study to assess dynamic connectivity properties in neonates. Compared to term neonates, the late preterm group shows a preference for a state with general weak connectivity and the length of time in that state is negatively correlated to the degree of maturity. We believe these findings provide new perspectives for understanding the state-dependent neurophysiological mechanisms in premature brain injury. However, due to unique challenges associated with neonatal neuroimaging acquisition and analysis, further targeted studies were needed in this high-risk population.

## Data Availability Statement

The datasets generated for this study are available on request to the corresponding author.

## Ethics Statement

The studies involving human participants were reviewed and approved by Ethics Committee of Daping Hospital, Army Medical University (Chongqing, China). Written informed consent to participate in this study was provided by the participants' legal guardian/next of kin.

## Author Contributions

YS and XM: study design, analysis, interpretation of data, drafting, and revising the article. XW: acquisition of data. YS final approval of the version to be published and providing funding for the study. All authors: contributed to the article and approved the submitted version.

## Conflict of Interest

The authors declare that the research was conducted in the absence of any commercial or financial relationships that could be construed as a potential conflict of interest.
